# The complete sequence of a human genome*

**DOI:** 10.1126/science.abj6987

**Published:** 2022-03-31

**Authors:** Sergey Nurk, Sergey Koren, Arang Rhie, Mikko Rautiainen, Andrey V. Bzikadze, Alla Mikheenko, Mitchell R. Vollger, Nicolas Altemose, Lev Uralsky, Ariel Gershman, Sergey Aganezov, Savannah J. Hoyt, Mark Diekhans, Glennis A. Logsdon, Michael Alonge, Stylianos E. Antonarakis, Matthew Borchers, Gerard G. Bouffard, Shelise Y. Brooks, Gina V. Caldas, Nae-Chyun Chen, Haoyu Cheng, Chen-Shan Chin, William Chow, Leonardo G. de Lima, Philip C. Dishuck, Richard Durbin, Tatiana Dvorkina, Ian T. Fiddes, Giulio Formenti, Robert S. Fulton, Arkarachai Fungtammasan, Erik Garrison, Patrick G.S. Grady, Tina A. Graves-Lindsay, Ira M. Hall, Nancy F. Hansen, Gabrielle A. Hartley, Marina Haukness, Kerstin Howe, Michael W. Hunkapiller, Chirag Jain, Miten Jain, Erich D. Jarvis, Peter Kerpedjiev, Melanie Kirsche, Mikhail Kolmogorov, Jonas Korlach, Milinn Kremitzki, Heng Li, Valerie V. Maduro, Tobias Marschall, Ann M. McCartney, Jennifer McDaniel, Danny E. Miller, James C. Mullikin, Eugene W. Myers, Nathan D. Olson, Benedict Paten, Paul Peluso, Pavel A. Pevzner, David Porubsky, Tamara Potapova, Evgeny I. Rogaev, Jeffrey A. Rosenfeld, Steven L. Salzberg, Valerie A. Schneider, Fritz J. Sedlazeck, Kishwar Shafin, Colin J. Shew, Alaina Shumate, Ying Sims, Arian F. A. Smit, Daniela C. Soto, Ivan Sović, Jessica M. Storer, Aaron Streets, Beth A. Sullivan, Françoise Thibaud-Nissen, James Torrance, Justin Wagner, Brian P. Walenz, Aaron Wenger, Jonathan M. D. Wood, Chunlin Xiao, Stephanie M. Yan, Alice C. Young, Samantha Zarate, Urvashi Surti, Rajiv C. McCoy, Megan Y. Dennis, Ivan A. Alexandrov, Jennifer L. Gerton, Rachel J. O’Neill, Winston Timp, Justin M. Zook, Michael C. Schatz, Evan E. Eichler, Karen H. Miga, Adam M. Phillippy

**Affiliations:** 1 Genome Informatics Section, Computational and Statistical Genomics Branch, National Human Genome Research Institute, National Institutes of Health; Bethesda, MD USA; 2 Graduate Program in Bioinformatics and Systems Biology, University of California, San Diego; La Jolla, CA, USA; 3 Center for Algorithmic Biotechnology, Institute of Translational Biomedicine, Saint Petersburg State University; Saint Petersburg, Russia; 4 Department of Genome Sciences, University of Washington School of Medicine; Seattle, WA, USA; 5 Department of Bioengineering, University of California, Berkeley; Berkeley, CA, USA; 6 Sirius University of Science and Technology; Sochi, Russia; 7 Vavilov Institute of General Genetics; Moscow, Russia; 8 Department of Molecular Biology and Genetics, Johns Hopkins University; Baltimore, MD, USA; 9 Department of Computer Science, Johns Hopkins University; Baltimore, MD, USA; 10 Institute for Systems Genomics and Department of Molecular and Cell Biology, University of Connecticut; Storrs, CT, USA; 11 UC Santa Cruz Genomics Institute, University of California, Santa Cruz; Santa Cruz, CA, USA; 12 University of Geneva Medical School; Geneva, Switzerland; 13 Stowers Institute for Medical Research; Kansas City, MO, USA; 14 NIH Intramural Sequencing Center, National Human Genome Research Institute, National Institutes of Health; Bethesda, MD, USA; 15 Department of Molecular and Cell Biology, University of California, Berkeley; Berkeley, CA, USA; 16 Department of Data Sciences, Dana-Farber Cancer Institute; Boston, MA; 17 Department of Biomedical Informatics, Harvard Medical School; Boston, MA; 18 DNAnexus; Mountain View, CA, USA; 19 Wellcome Sanger Institute; Cambridge, UK; 20 Department of Genetics, University of Cambridge; Cambridge, UK; 21 Inscripta; Boulder, CO, USA; 22 Laboratory of Neurogenetics of Language and The Vertebrate Genome Lab, The Rockefeller University; New York, NY, USA; 23 Howard Hughes Medical Institute; Chevy Chase, MD, USA; 24 Department of Genetics, Washington University School of Medicine; St. Louis, MO, USA; 25 University of Tennessee Health Science Center; Memphis, TN, USA; 26 McDonnell Genome Institute, Washington University in St. Louis; St. Louis, MO, USA; 27 Department of Genetics, Yale University School of Medicine; New Haven, CT, USA; 28 Comparative Genomics Analysis Unit, Cancer Genetics and Comparative Genomics Branch, National Human Genome Research Institute, National Institutes of Health; Bethesda, MD, USA; 29 Pacific Biosciences; Menlo Park, CA, USA; 30 Department of Computational and Data Sciences, Indian Institute of Science; Bangalore KA, India; 31 Reservoir Genomics LLC; Oakland, CA; 32 Department of Computer Science and Engineering, University of California, San Diego; San Diego, CA, USA; 33 Undiagnosed Diseases Program, National Human Genome Research Institute, National Institutes of Health; Bethesda, MD, USA; 34 Heinrich Heine University Düsseldorf, Medical Faculty, Institute for Medical Biometry and Bioinformatics; Düsseldorf, Germany; 35 Biosystems and Biomaterials Division, National Institute of Standards and Technology; Gaithersburg, MD, USA; 36 Department of Pediatrics, Division of Genetic Medicine, University of Washington and Seattle Children’s Hospital; Seattle, WA, USA; 37 Max-Planck Institute of Molecular Cell Biology and Genetics; Dresden, Germany; 38 Department of Psychiatry, University of Massachusetts Medical School; Worcester, MA, USA; 39 Faculty of Biology, Lomonosov Moscow State University; Moscow, Russia; 40 Cancer Institute of New Jersey; New Brunswick, NJ, USA; 41 Department of Biomedical Engineering, Johns Hopkins University; Baltimore, MD, USA; 42 National Center for Biotechnology Information, National Library of Medicine, National Institutes of Health; Bethesda, MD, USA; 43 Human Genome Sequencing Center, Baylor College of Medicine; Houston TX, USA; 44 Genome Center, MIND Institute, Department of Biochemistry and Molecular Medicine, University of California, Davis; CA, USA; 45 Institute for Systems Biology; Seattle, WA, USA; 46 Digital BioLogic d.o.o.; Ivanić-Grad, Croatia; 47 Chan Zuckerberg Biohub; San Francisco, CA, USA; 48 Department of Molecular Genetics and Microbiology, Duke University School of Medicine; Durham, NC, USA; 49 Department of Biology, Johns Hopkins University; Baltimore, MD, USA; 50 Department of Pathology, University of Pittsburgh; Pittsburgh, PA, USA; 51 Research Center of Biotechnology of the Russian Academy of Sciences; Moscow, Russia; 52 Department of Biochemistry and Molecular Biology, University of Kansas Medical School; Kansas City, MO, USA; 53 Department of Biomolecular Engineering, University of California Santa Cruz, CA, USA

## Abstract

Since its initial release in 2000, the human reference genome has covered only the euchromatic fraction of the genome, leaving important heterochromatic regions unfinished. Addressing the remaining 8% of the genome, the Telomere-to-Telomere (T2T) Consortium presents a complete 3.055 billion base pair (bp) sequence of a human genome, T2T-CHM13, that includes gapless assemblies for all chromosomes except Y, corrects errors in the prior references, and introduces nearly 200 million bp of sequence containing 1,956 gene predictions, 99 of which are predicted to be protein coding. The completed regions include all centromeric satellite arrays, recent segmental duplications, and the short arms of all five acrocentric chromosomes, unlocking these complex regions of the genome to variational and functional studies.

## One-Sentence Summary

Twenty years after the initial drafts, a truly complete sequence of a human genome reveals what has been missing.

The current human reference genome was released by the Genome Reference Consortium (GRC) in 2013 and most recently patched in 2019 (GRCh38.p13) (1). This reference traces its origin to the publicly funded Human Genome Project (2) and has been continually improved over the past two decades. Unlike the competing Celera effort (3) and most modern sequencing projects based on “shotgun” sequence assembly (4), the GRC assembly was constructed from sequenced bacterial artificial chromosomes (BACs) that were ordered and oriented along the human genome via radiation hybrid, genetic linkage, and fingerprint maps. However, limitations of BAC cloning led to an underrepresentation of repetitive sequences, and the opportunistic assembly of BACs derived from multiple individuals resulted in a mosaic of haplotypes. As a result, several GRC assembly gaps are unsolvable due to incompatible structural polymorphisms on their flanks, and many other repetitive and polymorphic regions were left unfinished or incorrectly assembled (5).

**Fig. 1.**
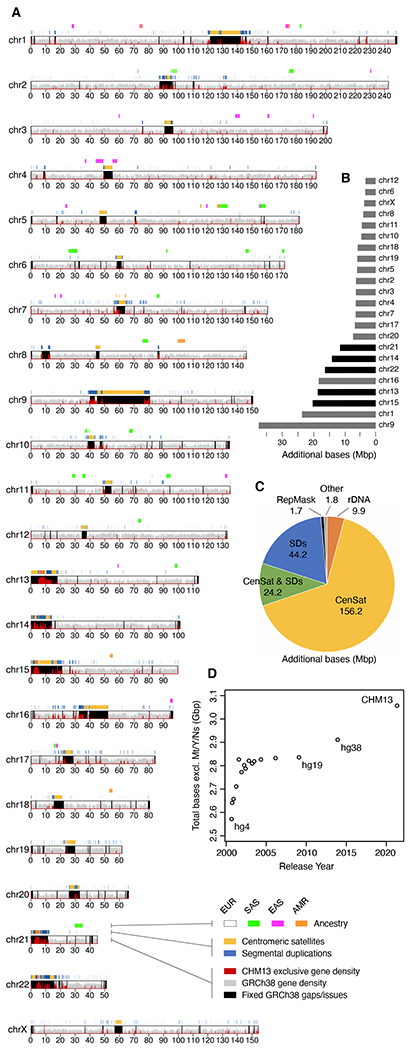
Summary of the complete T2T-CHM13 human genome assembly. (**A**) Ideogram of T2T-CHM13v1.1 assembly features. Bottom to top: gaps/issues in GRCh38 fixed by CHM13 overlaid with the density of genes exclusive to CHM13 in red; segmental duplications (SDs) (42) and centromeric satellites (CenSat) (30); and CHM13 ancestry predictions (EUR, European; SAS, South Asian; EAS, East Asian; AMR, Ad Mixed American). (**B**) Additional (non-syntenic) bases in the CHM13 assembly relative to GRCh38 per chromosome, with the acrocentrics highlighted in black, and (**C**) by sequence type (note that the CenSat and SD annotations overlap). (**D**) Total non-gap bases in UCSC reference genome releases dating back to September 2000 (hg4) and ending with T2T-CHM13 in 2021.

The GRCh38 reference assembly contains 151 Mbp of unknown sequence distributed throughout the genome, including pericentromeric and subtelomeric regions, recent segmental duplications, ampliconic gene arrays, and ribosomal DNA (rDNA) arrays, all of which are necessary for fundamental cellular processes (Fig. 1A). Some of the largest reference gaps include human satellite (HSat) repeat arrays and the short arms of all five acrocentric chromosomes, which are represented in GRCh38 as multi-megabase stretches of unknown bases (Figs. 1B and 1C). In addition to these apparent gaps, other regions of GRCh38 are artificial or are otherwise incorrect. For example, the centromeric alpha satellite arrays are represented as computationally generated models of alpha satellite monomers to serve as decoys for resequencing analyses (6), while sequence assigned to the short arm of Chromosome 21 appears falsely duplicated and poorly assembled (7). When compared to other human genomes, GRCh38 also shows a genome-wide deletion bias that is indicative of incomplete assembly (8). Despite finishing efforts from both the Human Genome Project (9) and GRC (1) that improved the quality of the reference, there was limited progress towards closing the remaining gaps in the years that followed (Fig. 1D).

Long-read shotgun sequencing overcomes the limitations of BAC-based assembly and bypasses the challenges of structural polymorphism between genomes. PacBio’s multi-kilobase, single-molecule reads (10) proved capable of resolving complex structural variation and gaps in GRCh38 (8, 11), while Oxford Nanopore’s >100 kbp “ultra-long” reads (12), enabled complete assemblies of a human centromere (ChrY) (13) and, later, an entire chromosome (ChrX) (14). However, the high error rate (>5%) of these technologies posed challenges for the assembly of long, near-identical repeat arrays. PacBio’s most recent “HiFi” circular consensus sequencing offers a compromise of 20 kbp read lengths with an error rate of 0.1% (15). Whereas ultra-long reads are useful for spanning repeats, HiFi reads excel at differentiating subtly diverged repeat copies or haplotypes (16).

To finish the last remaining regions of the genome, we leveraged the complementary aspects of PacBio HiFi and Oxford Nanopore ultra-long read sequencing to assemble the uniformly homozygous CHM13hTERT cell line (hereafter, CHM13) (17). The resulting T2T-CHM13 reference assembly removes a 20-year-old barrier that has hidden 8% of the genome from sequence-based analysis, including all centromeric regions and the entire short arms of five human chromosomes. Here we describe the construction, validation, and initial analysis of a truly complete human reference genome and discuss its potential impact on the field.

## Cell line and sequencing

As with many prior reference genome improvement efforts (1, 8, 17–20), including the T2T assemblies of human chromosomes X (14) and 8 (21), we targeted a complete hydatidiform mole for sequencing. Most CHM genomes arise from the loss of the maternal complement and duplication of the paternal complement postfertilization and are, therefore, homozygous with a 46,XX karyotype (22). Sequencing of CHM13 confirmed nearly uniform homozygosity, with the exception of a few thousand heterozygous variants and a megabase-scale heterozygous deletion within the rDNA array on Chromosome 15 (23) (figs. S1 to S2). Local ancestry analysis shows the majority of the CHM13 genome is of European origin, including regions of Neanderthal introgression, with some predicted admixture (23) (Fig. 1A). Compared to diverse samples from the 1000 Genomes Project (1KGP) (24), CHM13 possesses no apparent excess of singleton alleles or loss-of-function variants (25).

We extensively sequenced CHM13 with multiple technologies (23), including 30× PacBio circular consensus sequencing (HiFi) (16, 20), 120× Oxford Nanopore ultra-long read sequencing (ONT) (14, 21), 100× Illumina PCR-Free sequencing (ILMN) (1), 70× Illumina / Arima Genomics Hi-C (Hi-C) (14), BioNano optical maps (14), and Strand-seq (20) (table S1). To enable assembly of the highly repetitive centromeric satellite arrays and closely related segmental duplications, we developed methods for assembly, polishing, and validation that better utilize these available datasets.

## Genome assembly

The basis of the T2T-CHM13 assembly is a high-resolution assembly string graph (26) built directly from HiFi reads. In a bidirected string graph, nodes represent unambiguously assembled sequences and edges correspond to the overlaps between them, due to either repeats or true adjacencies in the underlying genome. The CHM13 graph was constructed using a purpose-built method that combines components from existing assemblers (16, 27) along with specialized graph processing (23). Most HiFi errors are small insertions or deletions within homopolymer runs and simple sequence repeats (16), so homopolymer runs were first “compressed” to a single nucleotide (e.g., [A]_n_ becomes [A]_1_ for *n* > 1). All compressed reads were then aligned to one another to identify and correct small errors, and differences within simple sequence repeats were masked. After compression, correction, and masking, only exact read overlaps were considered during graph construction, followed by iterative graph simplification (23).

**Fig. 2.**
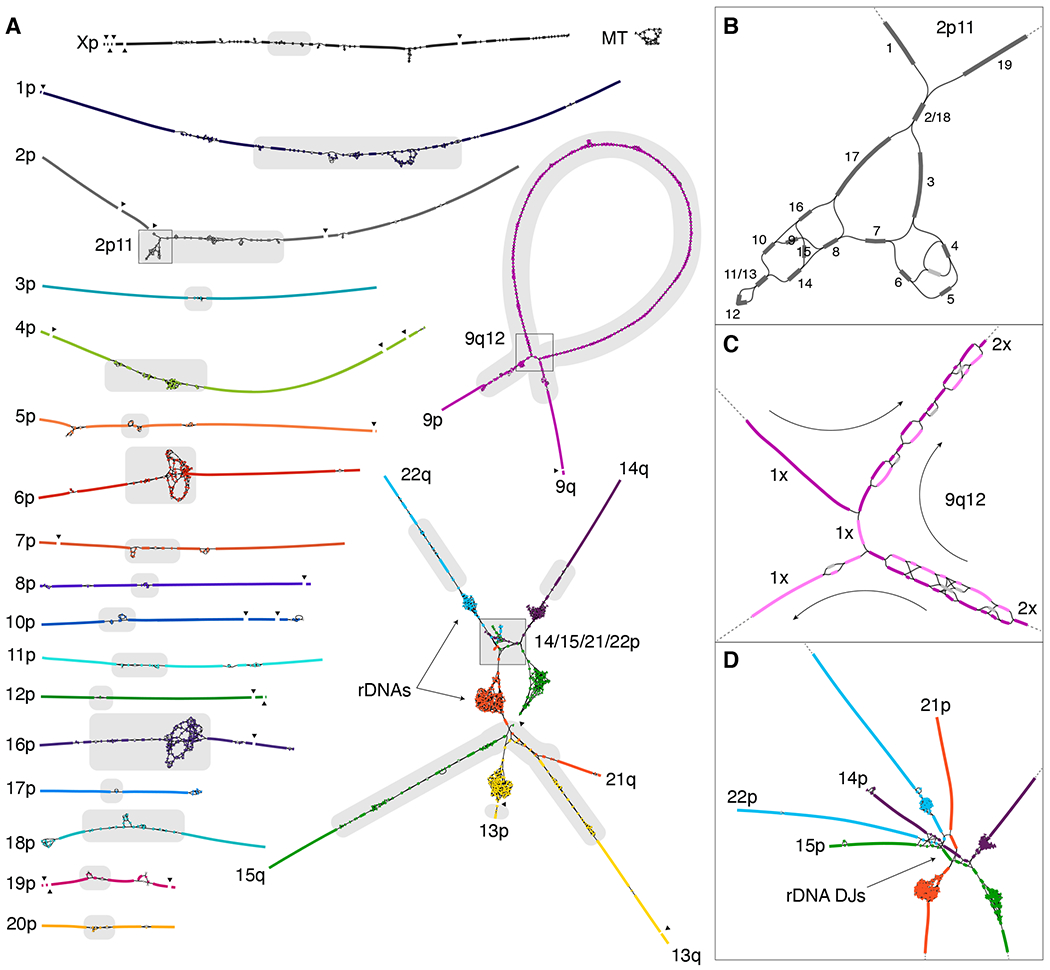
High-resolution assembly string graph of the CHM13 genome. (**A**) Bandage (60) visualization, where nodes represent unambiguously assembled sequences scaled by length, and edges correspond to the overlaps between node sequences. Each chromosome is both colored and numbered on the short (p) arm. Long (q) arms are labeled where unclear. The five acrocentric chromosomes (bottom right) are connected due to similarity between their short arms, and the rDNA arrays form five dense tangles due to their high copy number. The graph is partially fragmented due to HiFi coverage dropout surrounding GA-rich sequence (black triangles). Centromeric satellites (30) are the source of most ambiguity in the graph (gray highlights). (**B**) The ONT-assisted graph traversal for the 2p11 locus is given by numerical order. Based on low depth-of-coverage, the unlabeled light gray node represents an artifact or heterozygous variant and was not used. (**C**) The multi-megabase tandem HSat3 duplication (9qh+) at 9q12 requires two traversals of the large loop structure (the size of the loop is exaggerated because graph edges are of constant size). Nodes used by the first traversal are in dark purple and the second traversal in light purple. Nodes used by both traversals typically have twice the sequencing coverage. (**D**) Enlargement of the distal short arms of the acrocentrics, showing the colored graph walks and edges between highly similar sequences in the distal junctions (DJs) adjacent to the rDNA arrays.

In the resulting graph, most components originate from a single chromosome and have an almost linear structure (Fig. 2A), which suggests few perfect repeats greater than roughly 10 kbp exist between different chromosomes or distant loci. Two notable exceptions are the five acrocentric chromosomes, which form a single connected component in the graph, and a recent multi-megabase HSat3 duplication on Chromosome 9, consistent with the 9qh+ karyotype of CHM13 (fig. S3). Minor fragmentation of the chromosomes into multiple components resulted from a lack of HiFi sequencing coverage across GA-rich sequences (16). These gaps were later filled with a prior ONT-based assembly (CHM13v0.7) (14).

Ideally, the complete sequence for each chromosome should exist as a walk through the string graph where some nodes may be traversed multiple times (repeats) and some not at all (errors and heterozygous variants). To help identify the correct walks, we estimated coverage depth and multiplicity of the nodes (23), which allowed most tangles to be manually resolved as unique walks visiting each node the appropriate number of times (Figs. 2B and fig. S4). In the remaining cases, the correct path was ambiguous and required integration of ONT reads (Figs. 2C and 2D). Where possible, ONT reads were aligned to candidate traversals or directly to the HiFi graph (28) to guide the correct walk (fig. S5), but more elaborate strategies were required for recent satellite array duplications on chromosomes 6 and 9 (23). Only the five rDNA arrays, constituting approximately 10 Mbp of sequence, could not be resolved with the string graph and required a specialized approach (described below). An accurate consensus sequence for the selected graph walks was computed from the uncompressed HiFi reads (23), resulting in the CHM13v0.9 draft assembly.

For comparative genomics of the centromere (29, 30), we repeated this process on an additional X chromosome from the Coriell GM24385 cell line (NIST ID: HG002). The resulting T2T-HG002-ChrX assembly shows comparable accuracy to T2T-CHM13 (23) (figs. S6 to S8).

## rDNA assembly

The most complex region of the CHM13 string graph involves the human ribosomal DNA arrays and their surrounding sequence (Fig. 2D). Human rDNAs are 45 kbp near-identical repeats that encode the 45S rRNA and are arranged in large, tandem repeat arrays embedded within the short arms of the acrocentric chromosomes. The length of these arrays varies between individuals (36), and even somatically, especially with aging and certain cancers (37). A typical diploid human genome has an average of 315 rDNA copies, with a standard deviation of 104 copies (36). We estimate that the diploid CHM13 genome contains approximately 400 rDNA copies based on ILMN depth of coverage (23) (fig. S9), or 409 ± 9 (mean ± s.d.) rDNA copies by ddPCR (fig. S10).

To assemble these highly dynamic regions of the genome, and overcome limitations of the string graph construction (23) (fig. S11), we constructed sparse de Bruijn graphs for each of the five rDNA arrays (38) (fig. S12). ONT reads were aligned to the graphs to identify a set of walks, which were converted to sequence, segmented into individual rDNA units, and clustered into “morphs” according to their sequence similarity. The copy number of each morph was estimated from the number of supporting ONT reads, and consensus sequences were polished with mapped HiFi reads. ONT reads spanning two or more rDNA units were used to build a morph graph representing the structure of each array (fig. S12).

The shorter arrays on Chromosomes 14 and 22 consist of a single primary morph arranged in a head-to-tail array, whereas the longer arrays on Chromosomes 13, 15, and 21 exhibit a more mosaic structure involving multiple, interspersed morphs. In these cases, the ONT reads were not long enough to fully resolve the ordering, and the primary morphs were artificially arranged in consecutive blocks reflecting their estimated copy number. These three arrays capture the chromosome-specific morphs but should be treated as model sequences. The final T2T-CHM13 assembly contains 219 complete rDNA copies, totaling 9.9 Mbp of sequence.

## Assembly validation and polishing

To evaluate concordance between the reads and the assembly we mapped all available primary data, including HiFi, ONT, ILMN, Strand-seq, and Hi-C, to the CHM13v0.9 draft assembly to identify both small and structural variants (see reference (31) for a complete description). Manual curation corrected 4 large and 993 small errors, resulting in the CHM13v1.0 assembly, and identified 44 large and 3,901 small heterozygous variants (31). Further telomere polishing and addition of the rDNA arrays (23) resulted in a complete, telomere-to-telomere assembly of a human genome, T2T-CHM13v1.1.

The T2T-CHM13 assembly is consistent with previously validated assemblies of chromosomes X (14) and 8 (21), and the sizes of assembled satellite arrays match ddPCR copy-number estimates for those tested (fig. S10 and tables S2 and S3). Mapped Strand-seq (figs. S13 and S14) and Hi-C (fig. S15) data show no signs of misorientations or other large-scale structural errors. The assembly correctly resolves 644 of 647 previously sequenced CHM13 BACs at >99.99% identity, with the 3 others reflecting errors in the BACs themselves (figs. S16 to S19).

**Fig. 3.**
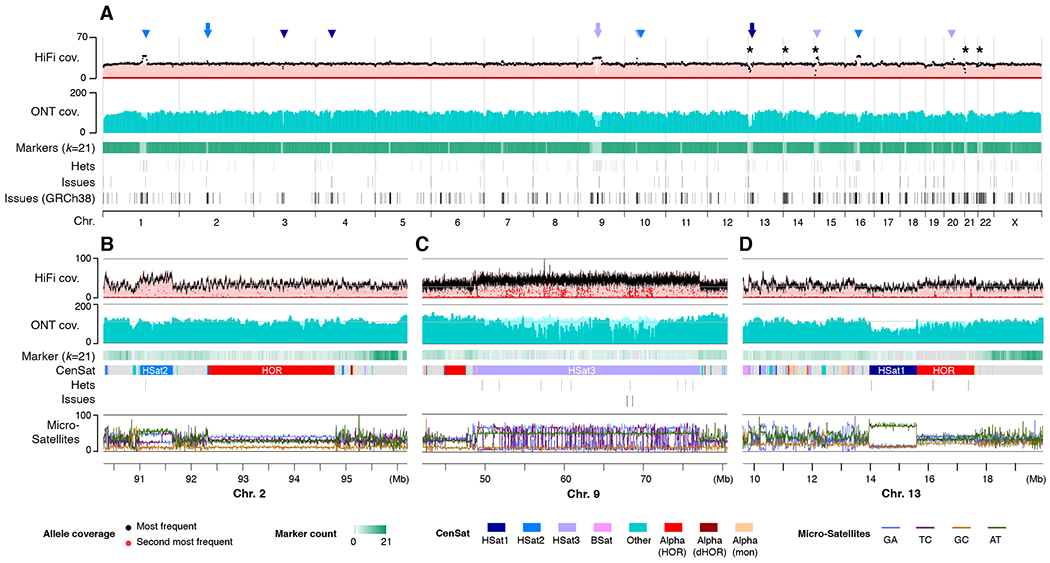
Sequencing coverage and assembly validation. (**A**) Uniform whole-genome coverage of mapped HiFi and ONT reads is shown with primary alignments in light shades and marker-assisted alignments overlaid in dark shades. Large HSat arrays (30) are noted by triangles, with inset regions are marked by arrowheads and the location of the rDNA arrays marked with asterisks. Regions with low unique marker frequency (light green) correspond to drops in unique marker density, but are recovered by the lower-confidence primary alignments. Annotated assembly issues are compared for T2T-CHM13 and GRCh38. (**B–D**) Enlargements corresponding to regions of the genome featured in Fig. 2. Uniform coverage changes within certain satellites are reproducible and likely caused by sequencing bias. Identified heterozygous variants and assembly issues are marked below and typically correspond with low coverage of the primary allele (black) and elevated coverage of the secondary allele (red). % microsatellite repeats for every 128 bp window is shown at the bottom.

Mapped sequencing read depth shows uniform coverage across all chromosomes (Fig. 3A), with 99.86% of the assembly within three standard deviations of the mean coverage for either HiFi or ONT (HiFi coverage 34.70 ± 7.03, ONT coverage 116.16 ± 16.96, excluding the mitochondrial genome). Ignoring the 10 Mbp of rDNA sequence, where most of the coverage deviation resides, 99.99% of the assembly is within three standard deviations (23). Alignment-free analysis of ILMN and HiFi copy number data also show concordance with the assembly (figs. S20 and S21). This is consistent with uniform coverage of the genome and confirms both the accuracy of the assembly and the absence of aneuploidy in the sequenced CHM13 cells.

Coverage increases or decreases were observed across multiple satellite arrays (Figs. 3B to 3D). However, given the uniformity of coverage across these arrays, association with specific satellite classes, and the sometimes opposite effect observed for HiFi and ONT, we hypothesize that these anomalies are related to biases introduced during sample preparation, sequencing, or basecalling, rather than assembly error (23) (figs. S22 to S26 and table S4). While the specific mechanisms require further investigation, prior studies have noted similar biases within certain satellite arrays and sequence contexts for both ONT and HiFi (32, 33).

Being the most difficult regions of the genome to assemble, we performed targeted validation of long tandem repeats to identify any errors missed by the genome-wide approach. The assembled rDNA morphs, being only 45 kbp each, were manually validated via inspection of the read alignments used for polishing. Alpha satellite higher-order repeats (HOR) were validated using a purpose-built method (34) (fig. S27 and table S5) and compared to independent ILMN-based HOR copy number estimates (fig. S28). All centromeric satellite arrays, including beta satellite (BSat) and human satellite (HSat) repeats, were further validated by measuring the ratio of primary to secondary variants identified by HiFi reads (35) (fig. S29).

The consensus accuracy of the T2T-CHM13 assembly is estimated to be approximately 1 error per 10 Mbp (23, 31), which exceeds the historical standard of “finished” sequence by orders of magnitude. However, regions of low HiFi coverage were found to be associated with an enrichment of potential errors, as estimated from both HiFi and ILMN data (31). To guide future use of the assembly, we have cataloged all low-coverage, low-confidence, and known heterozygous sites identified by the above validation procedures (31). The total number of bases covered by potential issues in the T2T-CHM13 assembly is just 0.3% of the total assembly length compared to 8% for GRCh38 (Fig. 3A).

## A truly complete genome

T2T-CHM13 includes gapless telomere-to-telomere assemblies for all 22 human autosomes and Chromosome X, comprising 3,054,815,472 bp of nuclear DNA, plus a 16,569 bp mitochondrial genome. This complete assembly adds or corrects 238 Mbp of sequence that does not co-linearly align to GRCh38 over a 1 Mbp interval (i.e., is non-syntenic), primarily comprising centromeric satellites (76%), non-satellite segmental duplications (19%), and rDNAs (4%) (Fig. 1C). 182 Mbp of sequence has no primary alignments to GRCh38 and is exclusive to T2T-CHM13. As a result, T2T-CHM13 increases the number of known genes and repeats in the human genome (Table 1).

To provide an initial annotation, we used both the Comparative Annotation Toolkit (CAT) (39) and Liftoff (40) to project the GENCODE v35 (41) reference annotation onto the T2T-CHM13 assembly. Additionally, CHM13 Iso-Seq transcriptome reads were assembled into transcripts and provided as complementary input to CAT. A comprehensive annotation was built by combining the CAT annotation with genes identified only by Liftoff (23).

The draft T2T-CHM13 annotation totals 63,494 genes and 233,615 transcripts, of which 19,969 genes (86,245 transcripts) are predicted to be protein coding, with 683 predicted frameshifts in 385 genes (469 transcripts) (Table 1, fig. S30, tables S6 to S8). Only 263 GENCODE genes (448 transcripts) are exclusive to GRCh38 and have no assigned ortholog in the CHM13 annotation (tables S9 and S10). Of these, 194 are due to a lower copy number in the CHM13 annotation (fig. S31), 46 do not align well to CHM13, and 23 correspond to known false-duplications in GRCh38 (25) (fig. S32). The majority of these genes are non-coding and associated with repetitive elements. Only 4 are annotated as being medically relevant (*CFHR1*, *CFHR3*, *OR51A2*, *UGT2B28*), all of which are due to lower copy number, and the only protein coding genes that align poorly are immunoglobulin and T-cell receptor genes, which are known to be highly diverse.

In comparison, a total of 3,604 genes (6,693 transcripts) are exclusive to CHM13 (tables S11 and S12). Most of these genes represent putative paralogs and localize to pericentromeric regions and the short arms of the acrocentrics, including 876 rRNA transcripts. Only 48 of the CHM13-exclusive genes (56 transcripts) were predicted solely from de novo assembled transcripts. Of all genes exclusive to CHM13, 140 are predicted to be protein coding based on their GENCODE paralogs and have a mean of 99.5% nucleotide and 98.7% amino acid identity to their most similar GRCh38 copy (table S13). While some of these additional paralogs may be present (but unannotated) in GRCh38 (23), 1,956 of the genes exclusive to CHM13 (99 protein coding) are in regions with no primary alignment to GRCh38 (table S11). A broader set of 182 multi-exon protein coding genes fall within non-syntenic regions, 36% of which were confirmed to be expressed in CHM13 (42).

Compared to GRCh38, T2T-CHM13 is a more complete, accurate, and representative reference for both short- and long-read variant calling across human samples of all ancestries (25). Reanalysis of 3,202 short-read datasets from the 1KGP showed that T2T-CHM13 simultaneously reduces both false-negative and false-positive variant calls due to the addition of 182 Mbp of missing sequence and the exclusion of 1.2 Mbp of falsely duplicated sequence in GRCh38. These improvements, combined with a lower frequency of rare variants and errors in T2T-CHM13, eliminate tens of thousands of spurious variants per 1KGP sample (25). In addition, the T2T-CHM13 reference was found to be more representative of human copy number variation than GRCh38 when compared against 268 human genomes from the Simons Genome Diversity Project (SGDP) (42, 43). Specifically, within non-syntenic segmentally duplicated regions of the genome, T2T-CHM13 is nine times more predictive of SGDP copy number than GRCh38 (42). These results underscore both the quality of the assembly and the genomic stability of the cell line from which it was derived.

## Acrocentric chromosomes

**Fig. 4.**
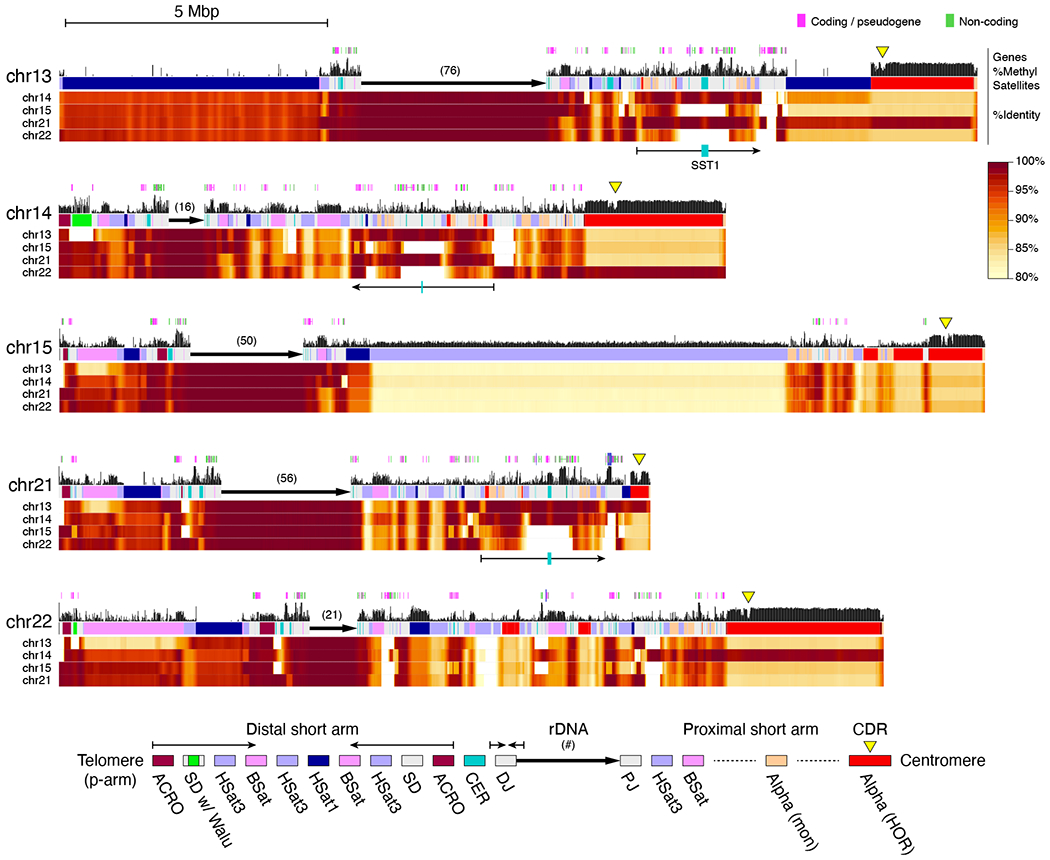
Short arms of the acrocentric chromosomes. Each short arm is shown along with annotated genes, percent of methylated CpGs (29), and a color-coded satellite repeat annotation (30). The rDNA arrays are represented by a directional arrow and copy number due to their high self-similarity, which prohibits ONT mapping. Percent identity heatmaps versus the other four arms were computed in 10 kbp windows and smoothed over 100 kbp intervals. Each position shows the maximum identity of that window to any window in the other chromosome. The distal short arms include conserved satellite structure and inverted repeats (thin arrows), while the proximal short arms show a diversity of structures. The proximal short arms of Chromosomes 13, 14, and 21 share a segmentally duplicated core, including small alpha satellite HOR arrays and a central, highly methylated, SST1 array (thin arrows with teal block). Yellow triangles indicate hypomethylated centromeric dip regions (CDRs), marking the sites of kinetochore assembly (29).

T2T-CHM13 uncovers the genomic structure of the short arms of the five acrocentric chromosomes, which, despite their importance for cellular function (44), have remained largely unsequenced to date. This omission has been due to their enrichment for satellite repeats and segmental duplications, which has prohibited sequence assembly and limited their characterization to cytogenetics, restriction mapping, and BAC sequencing (45–47). All five of CHM13’s short arms follow a similar structure consisting of an rDNA array embedded within distal and proximal repeat arrays (Fig. 4). From telomere to centromere, the short arms vary in size from 10.1 Mbp (Chr14) to 16.7 Mbp (Chr15), with a combined length of 66.1 Mbp.

Compared to other human chromosomes, the short arms of the acrocentrics are unusually similar to one another. Specifically, we find that 5 kbp windows align with a median identity of 98.7% between the short arms, creating many opportunities for interchromosomal exchange (Fig. 4). This high degree of similarity is presumably due to recent non-allelic or ectopic recombination stemming from their colocalization in the nucleolus (46). Additionally, considering an 80% identity threshold, no 5 kbp window on the short arms is unique and 96% of the non-rDNA sequence can be found elsewhere in the genome, suggesting the acrocentrics are dynamic sources of segmental duplication.

CHM13’s rDNA arrays vary in size from 0.7 Mbp (Chr14) to 3.6 Mbp (Chr13) and are in the expected arrangement, organized as head-to-tail tandem arrays with all 45S transcriptional units pointing towards the centromere. No inversions were noted within the arrays and nearly all rDNA units are full length, in contrast to some prior studies that reported embedded inversions and other non-canonical structures (47, 48). Each array appears highly homogenized, and there is more variation between rDNA units on different chromosomes than within chromosomes (fig. S33), suggesting that intra-chromosomal exchange of rDNA units via non-allelic homologous recombination is more common than inter-chromosomal exchange.

Many 45S gene copies on the same chromosome are identical to one another, while the identity of the most frequent 45S morphs between chromosomes ranges from 99.4–99.7%. A Chromosome 15 rDNA morph shows the highest identity (98.9%) to the current KY962518.1 rDNA reference sequence, originally derived from a human Chromosome 21 BAC clone (47). As expected, the 13 kbp 45S is more conserved than the intergenic spacer (IGS), with all major 45S morphs aligning between 99.4–99.6% identity to KY962518.1. Certain rDNA variants appear chromosome-specific, including single-nucleotide variants within the 45S and its upstream promoter region (fig. S34). The most evident variants are repeat expansions and contractions within the tandem “R” repeat that immediately follows the 45S and the CT-rich “Long” repeat located in the middle of the IGS. The most frequent morph in each array can be uniquely distinguished by these two features (fig. S35).

From the telomere to the rDNA array, the structure of all five distal short arms follows a similar pattern involving a symmetric arrangement of inverted segmental duplications and ACRO, HSat3, BSat, and HSat1 repeats (Fig. 4); however, the sizes of these repeat arrays varies among chromosomes. Chromosome 13 is missing the distal half of the inverted duplication and has an expanded HSat1 array relative to the others. Despite their variability in size, all satellite arrays share a high degree of similarity (typically >90% identity) both within and between acrocentric chromosomes. Chromosomes 14 and 22 also feature the expansion of a 64-bp Alu-associated satellite repeat (“Walu”) within the distal inverted duplication (49), the location of which was confirmed via FISH (fig. S36). The distal junction (DJ) immediately prior to the rDNA array includes centromeric repeats (CER) and a highly conserved and actively transcribed 200 kbp palindromic repeat, which agrees with previous characterizations of the rDNA flanking sequences (46, 50).

Extending from the rDNA array to the centromere, the proximal short arms are larger in size and show a higher diversity of structures including shuffled segmental duplications (42), composite transposable element arrays (49), satellite arrays (including HSat3, BSat, HSat1, HSat5), and alpha satellite arrays (both monomeric and HORs) (30). Some proximal BSat arrays show a mosaic inversion structure that was also observed in HSat arrays elsewhere in the genome (30) (fig. S37). The proximal short arms of chromosomes 13, 14, and 21 appear to share the highest degree of similarity with a large region of segmental duplication including similar HOR subsets and a central and highly methylated SST1 array (Fig. 4). This coincides with these three chromosomes being most frequently involved in Robertsonian translocations (51). Alpha satellite HORs on chromosomes 13/21 and chromosomes 14/22 also share high similarity within each pair, but not between them (52, 53). Non-satellite sequences within these segmental duplications often exceed 99% identity and show evidence of transcription (29, 42, 49). Using the T2T-CHM13 reference as a basis, further study of additional genomes is now needed to understand which of these features are conserved across the human population.

## Analyses and resources

A number of companion studies were carried out to characterize the complete sequence of a human genome, including comprehensive analyses of centromeric satellites (30), segmental duplications (42), transcriptional (49) and epigenetic profiles (29), mobile elements (49), and variant calls (25). Up to 99% of the complete CHM13 genome can be confidently mapped with long-read sequencing, opening these regions of the genome to functional and variational analysis (23) (fig. S38 and table S14). We have produced a rich collection of annotations and omics datasets for CHM13, including RNA-Seq (30), Iso-Seq (21), PRO-Seq (49), CUT&RUN (30), and ONT methylation (29) experiments, and have made these datasets available via a centralized UCSC Assembly Hub genome browser (54).

To highlight the utility of these genetic and epigenetic resources mapped to a complete human genome, we provide the example of a segmentally duplicated region of the Chromosome 4q subtelomere that is associated with facioscapulohumeral muscular dystrophy (FSHD) (55). This region includes FSHD region gene 1 (*FRG1*), FSHD region gene 2 (*FRG2*), and an intervening D4Z4 macrosatellite repeat containing the double homeobox 4 (*DUX4*) gene that has been implicated in the etiology of FSHD (56). Numerous duplications of this region throughout the genome have complicated past genetic analyses of FSHD.

**Fig. 5.**
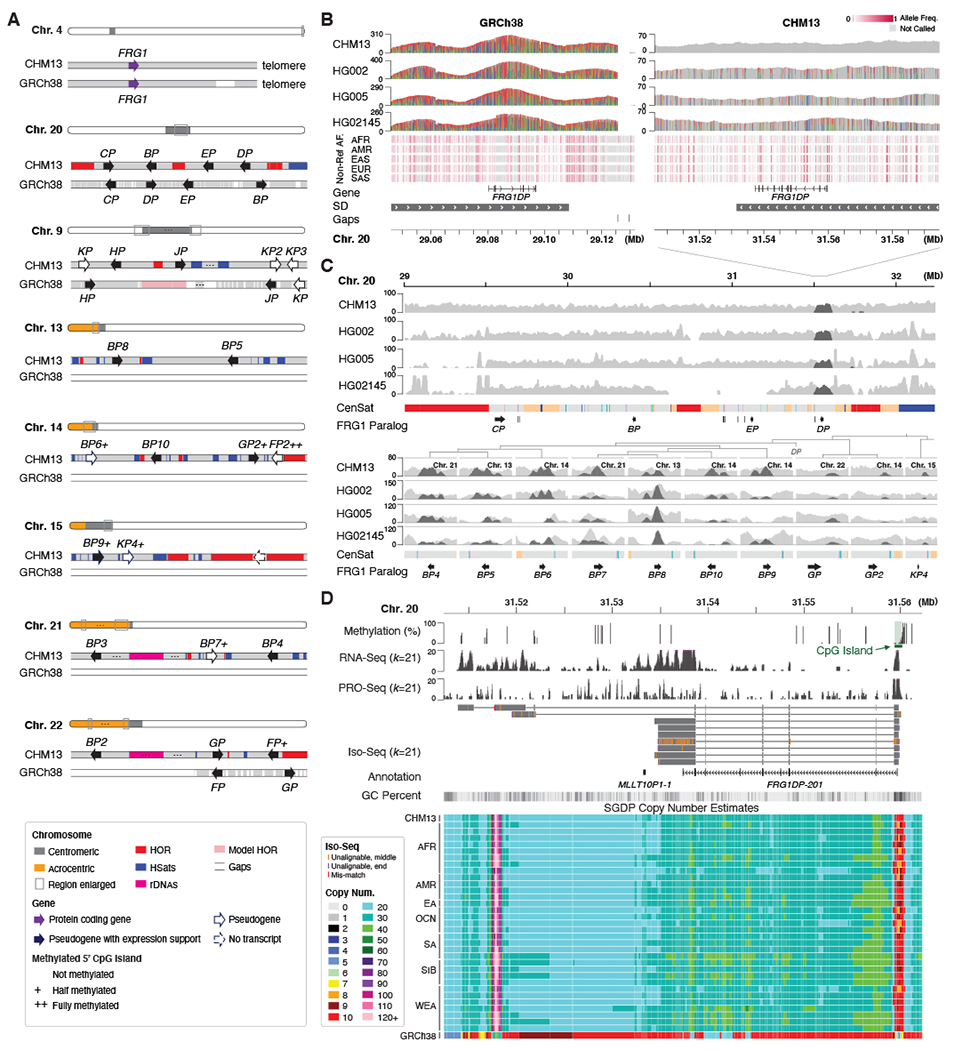
Resolved FRG1 paralogs. (**A**) Protein-coding gene *FRG1* and its 23 paralogs in CHM13. Only 9 are found in GRCh38. Genes are drawn larger than their actual size and the “FRG1” prefix is omitted for brevity. All paralogs are found near satellite arrays. Most copies exhibit evidence of expression, including CpG islands present at the 5′ start site with varying degrees of methylation. (**B**) Reference (gray) and variant (colored) allele coverage is shown for four human HiFi samples mapped to the paralog *FRG1DP*. When mapped to GRCh38, the region shows excessive HiFi coverage and variants, indicating that reads from the missing paralogs are mis-mapped to *FRG1DP* (variants with >80% coverage shown). When mapped to CHM13, HiFi reads show the expected coverage and a typical heterozygous variation pattern for the three non-CHM13 samples (variants >20% coverage shown). These non-reference alleles are also found in other populations from 1KGP ILMN data. (**C**) Mapped HiFi read coverage for other *FRG1* paralogs, with an extended context shown for Chromosome 20. Coverage of HiFi reads that mapped to *FRG1DP* in GRCh38 are highlighted (dark gray), showing the paralogous copies they originate from (*FRG1BP4–10*, *FRG1GP*, *FRG1GP2*, and *FRG1KP4*). Background coverage is variable for some paralogs, suggesting copy number polymorphism in the population. (**D**) Methylation and expression profiles suggest transcription of *FRG1DP* in CHM13. In the copy number display (bottom), each length *k* sequence (*k*-mer) of the CHM13 assembly is painted with a color representing the copy number of that *k*-mer sequence in an SGDP sample. The CHM13 and GRCh38 tracks show the copy number of these same *k*-mers in the respective assemblies. CHM13 copy number resembles all samples from the SGDP, whereas GRCh38 underrepresents the true copy number.

The T2T-CHM13 assembly reveals 23 paralogs of *FRG1* spread across all acrocentric chromosomes as well as chromosomes 9 and 20 (Fig. 5A). This gene appears to have undergone recent amplification in the great apes (57), and approximate locations of *FRG1* paralogs were previously identified by fluorescence in situ hybridization (58). However, only 9 *FRG1* paralogs are found in GRCh38, hampering sequence-based analysis.

One of the few *FRG1* paralogs included in GRCh38, *FRG1DP*, is located in the centromeric region of Chromosome 20 and shares high identity (97%) with several paralogs (*FRG1BP4*–*10*) (23) (fig. S39 and tables S15 and S16). When mapping HiFi reads, absence of the additional *FRG1* paralogs in GRCh38 causes their reads to incorrectly align to *FRG1DP* resulting in many false-positive variants (Fig. 5B). Most *FRG1* paralogs appear present in other human genomes (Fig. 5C), and all except *FRG1KP2* and *FRG1KP3* have upstream CpG islands and some degree of expression evidence in CHM13 (Fig. 5D, table S17). Any variants within these paralogs, and others like them, will be overlooked when using GRCh38 as a reference.

## Future of the human reference genome

The T2T-CHM13 assembly adds five full chromosome arms and more additional sequence than any genome reference release in the past 20 years (Fig. 1D). This 8% of the genome has not been overlooked due to its lack of importance, but rather due to technological limitations. High accuracy long-read sequencing has finally removed this technological barrier, enabling comprehensive studies of genomic variation across the entire human genome, which we expect to drive future discovery in human genomic health and disease. Such studies will necessarily require a complete and accurate human reference genome.

CHM13 lacks a Y chromosome, and homozygous Y-bearing CHMs are non-viable, so a different sample type will be required to complete this last remaining chromosome. However, given its haploid nature, it should be possible to assemble the Y chromosome from a male sample using the same methods described here, and supplement the T2T-CHM13 reference assembly with a Y chromosome as needed.

Extending beyond the human reference genome, large-scale resequencing projects have revealed genomic variation across human populations. Our reanalyses of 1KGP (25) and SGDP (42) datasets have already shown the advantages of T2T-CHM13, even for short-read analyses. However, these studies give only a glimpse of the extensive structural variation that lies within the most repetitive regions of the genome assembled here. Long-read resequencing studies are now needed to comprehensively survey polymorphic variation and reveal any phenotypic associations within these regions.

Although CHM13 represents a complete human haplotype, it does not capture the full diversity of human genetic variation. To address this bias, the Human Pangenome Reference Consortium (HPRC) (59) has joined with the T2T Consortium to build a collection of high-quality reference haplotypes from a diverse set of samples. Ideally, all genomes could be assembled at the quality achieved here, but automated T2T assembly of diploid genomes presents a difficult challenge that will require continued development. Until this goal is realized, and any human genome can be completely sequenced without error, the T2T-CHM13 assembly represents a more complete, representative, and accurate reference than GRCh38.

## Supplementary Material

supp material

supp tables

## Figures and Tables

**Table 1.** Comparison of GRCh38 and T2T-CHM13v1.1 human genome assemblies.

Summary	GRCh38	T2T-CHM13	±%
Assembled bases (Gbp)	2.92	3.05	+4.5%
Unplaced bases (Mbp)	11.42	0	−100.0%
Gap bases (Mbp)	120.31	0	−100.0%
# Contigs	949	24	−97.5%
Ctg NG50 (Mbp)	56.41	154.26	+173.5%
# Issues	230	46	−80.0%
Issues (Mbp)	230.43	8.18	−96.5%

**Gene Annotation**			

# Genes	60,090	63,494	+5.7%
protein coding	19,890	19,969	+0.4%
# Exclusive genes	263	3,604	
protein coding	63	140	
# Transcripts	228,597	233,615	+2.2%
protein coding	84,277	86,245	+2.3%
# Exclusive transcripts	1,708	6,693	
protein coding	829	2,780	

**Segmental duplications (SDs)**			

% SDs	5.00%	6.61%	
SD bases (Mbp)	151.71	201.93	+33.1%
# SDs	24097	41528	+72.3%

**RepeatMasker**			

% Repeats	51.89%	53.94%	
Repeat bases (Mbp)	1,516.37	1,647.81	+8.7%
LINE	626.33	631.64	+0.8%
SINE	386.48	390.27	+1.0%
LTR	267.52	269.91	+0.9%
Satellite	76.51	150.42	+96.6%
DNA	108.53	109.35	+0.8%
Simple repeat	36.5	77.69	+112.9%
Low complexity	6.16	6.44	+4.6%
Retroposon	4.51	4.65	+3.3%
rRNA	0.21	1.71	+730.4%

GRCh38 summary statistics exclude “alts” (110 Mbp), patches (63 Mbp), and Chromosome Y (58 Mbp). Assembled bases: all non-N bases. Unplaced bases: not assigned or positioned within a chromosome. # Contigs: GRCh38 scaffolds were split at three consecutive Ns to obtain contigs. NG50: half of the 3.05 Gbp human genome size contained in contigs of this length or greater. # Exclusive genes/transcripts: for GRCh38, GENCODE genes/transcripts not found in CHM13; for CHM13, extra putative paralogs that are not in GENCODE. Segmental duplication analysis is from (42). RepeatMasker analysis is from (49).
